# Ciliate diversity and distribution patterns in the sediments of a seamount and adjacent abyssal plains in the tropical Western Pacific Ocean

**DOI:** 10.1186/s12866-017-1103-6

**Published:** 2017-09-12

**Authors:** Feng Zhao, Sabine Filker, Thorsten Stoeck, Kuidong Xu

**Affiliations:** 1 Department of Marine Organism Taxonomy and Phylogeny, Institute of Oceanology, Chinese Academy of Sciences, 7 Nanhai Road, Qingdao, 266071 People’s Republic of China; 2 Department of Molecular Ecology, University of Kaiserslautern, 67663 Kaiserslautern, Germany; 3 Department of Ecology, University of Kaiserslautern, 67663 Kaiserslautern, Germany; 4 University of Chinese Academy of Sciences, Beijing, 100049 China

**Keywords:** Deep sea, Seamount, Protozoa, Depth gradient, Vertical distribution, Ciliate

## Abstract

**Background:**

Benthic ciliates and the environmental factors shaping their distribution are far from being completely understood. Likewise, deep-sea systems are amongst the least understood ecosystems on Earth. In this study, using high-throughput DNA sequencing, we investigated the diversity and community composition of benthic ciliates in different sediment layers of a seamount and an adjacent abyssal plain in the tropical Western Pacific Ocean with water depths ranging between 813 m and 4566 m. Statistical analyses were used to assess shifts in ciliate communities across vertical sediment gradients and water depth.

**Results:**

Nine out of 12 ciliate classes were detected in the different sediment samples, with Litostomatea accounting for the most diverse group, followed by Plagiopylea and Oligohymenophorea. The novelty of ciliate genetic diversity was extremely high, with a mean similarity of 93.25% to previously described sequences. On a sediment depth gradient, ciliate community structure was more similar within the upper sediment layers (0-1 and 9-10 cm) compared to the lower sediment layers (19-20 and 29-30 cm) at each site. Some unknown ciliate taxa which were absent from the surface sediments were found in deeper sediments layers. On a water depth gradient, the proportion of unique OTUs was between 42.2% and 54.3%, and that of OTUs shared by all sites around 14%. However, alpha diversity of the different ciliate communities was relatively stable in the surface layers along the water depth gradient, and about 78% of the ciliate OTUs retrieved from the surface layer of the shallowest site were shared with the surface layers of sites deeper than 3800 m. Correlation analyses did not reveal any significant effects of measured environmental factors on ciliate community composition and structure.

**Conclusions:**

We revealed an obvious variation in ciliate community along a sediment depth gradient in the seamount and the adjacent abyssal plain and showed that water depth is a less important factor shaping ciliate distribution in deep-sea sediments unlike observed for benthic ciliates in shallow seafloors. Additionally, an extremely high genetic novelty of ciliate diversity was found in these habitats, which points to a hot spot for the discovery of new ciliate species.

**Electronic supplementary material:**

The online version of this article (10.1186/s12866-017-1103-6) contains supplementary material, which is available to authorized users.

## Background

Deep-sea systems (> 1000 m) cover more than 65% of the Earth’s surface and fulfill a range of key ecosystem functions [1]. Yet, they are amongst the least understood ecosystems on Earth [2]. Among various deep-sea habitats, seamounts are widespread and prominent features of the world’s underwater landscape [3, 4]. Their specific topography creates distinct habitats, characterized by particular hydrography and substrate types, which influence the diversity of the benthos [5]. So far, most biological research related to seamounts focused on patterns of mega- and macrobenthic biodiversity and their biogeography [4]. Information about the diversity and distribution of benthic protists from seamount sediments are scarce, with the exception of benthic foraminifera, whose seamount communities have been described by several authors using morphological criteria [6]. In the past decade, molecular techniques and sequencing the 18S rDNA as a taxonomic marker were used to investigate the diversity of protists in deep-sea sediments. These studies did not only reveal high protistan diversities in various deep-sea habitats [7, 8], but also numerous undescribed taxa and even several early branching eukaryotic lineages [9]. In some of these habitats, ciliates were the predominant and often the most diverse microeukaryotic group [10–13]. However, knowledge on their distribution patterns and the major factors enforcing their dispersal in deep-sea sediments are far from complete.

In a microscopy study, water depth emerged as an important factor structuring the distribution of benthic protists, including ciliates, in the deep sea [14]. However, because protists are not highly abundant in the deep sea [15], it is not surprising that microscopy studies miss a large proportion of the deep-sea protist diversity. Accordingly, using molecular techniques, more than 125 ciliate OTUs were detected in less than 1 g of abyssal sediments [7]. This exceeded the number of ciliate OTUs detected in the coastal sediments [16]. Thus, it is reasonable to assume that a large proportion of ciliate diversity in the deep-sea is still unknown to science.

Moreover, studies of benthic protists mainly focused on the top 1-2 cm surface layer of deep-sea sediments [7]. However, it is well known that different ciliate species are not equally distributed along a vertical sediment gradient, mostly owed to oxygen, organic carbon and grain-size gradients [17–20]. Thus, some unknown ciliate taxa which are absent from the surface sediments are likely to be found in deeper sediment layers.

In this study, we collected sediment samples from four sites in the tropical Western Pacific Ocean with water depths ranging between 813 m and 4566 m as well as from different sediment layers. This sampling strategy was to maximize the number of different deep-sea habitats and thus, the proportion of novel ciliate diversity to be found. These habitats included a seamount and the adjacent abyssal plain. Statistical analyses were then used to assess shifts in ciliate communities across vertical sediment gradients and water depth.

## Results

### Environmental parameters of sampling sites

For each sediment sample, the median grain size, the proportion of each sediment component and the total organic carbon (TOC) content were analyzed. The median grain size (mgs) of sediments differed notably among the different layers and sites and ranged between 4.5 * 10^−13^ μm (DS2.2) and 799.4 μm (S-B.1; Table 1). The mgs of surface sediments was highest at all sampling sites, but site DS1. Here, the highest mgs was detected at 20 cm depth. Sediment composition was different for each sampling site (Table 1). The TOC content of the sediments at sites S-M, S-B and DS1 varied between 0.1% (S-M.1) and 0.82% (DS1.3). The TOC content at site DS2 was, in contrast, more than 8 times higher than at the other sampling sites (e.g. DS2.2: 7.95%; Table 1).

**Table 1:** Environmental parameters of each sample (S = sand, St = silt, C = clay)

	Layer (cm)	Depth (m)	Longitude	Latitude	Total organic Carbon (%)	Median grain Size (μm)	Sand (%)	Silt (%)	Clay (%)	Type of sediments
S-M.1	0-1 cm	813	137°45.3′E	8°52.2′N	0.10	236.2	88.7	9.0	2.3	S
S-M.2	9-10 cm				0.14	73.8	53.8	30.8	15.4	S-St
S-M.3	19-20 cm				0.53	45.2	39.2	42.9	17.9	St-S
S-M.4	29-30 cm				0.06	42.4	39.4	44.8	15.8	St-S
S-B.1	0-1 cm	3812	137°54.4′E	8°47.0′N	0.63	799.4	62.7	23.3	9.1	S
S-B.2	9-10 cm				0.26	8.4	5.7	62.5	31.8	C-St
S-B.3	19-20 cm				0.41	25.3	26.1	53.8	20.2	S-C-St
DS1.1	0-1 cm	4042	134°50.1′E	10°0.3 ‘N	0.52	21.6	15.4	67.9	16.7	C-St
DS1.2	9-10 cm				0.42	33.7	39.7	55.3	5.1	S-St
DS1.3	19-20 cm				0.82	85.2	63.6	33.3	3.1	St-S
DS2.1	0-1 cm	4566	136 °0.2′E	9 °0.3′N	7.14	18.1	3.3	78.3	18.5	C-St
DS2.2	9-10 cm				7.95	4.5 * 10^−13^	0.0	75.3	24.7	C-St

### Overview of the sequencing data

For the 12 sediment samples analyzed in this study, we obtained a total of 640,860 high-quality ciliate V4 sequences, which clustered into 104 distinct operational taxonomic units (OTUs) based on a 97% sequence similarity. The number of reads per sample varied between 25,169 (DS1.2) and 137,990 (S-B.2), with an average of 56,820 reads. The total number of OTUs obtained from each sample and used in downstream analyses ranged between 12 (S-B.2) and 55 (S-M.3), with an average of 33 OTUs (Table 2). Rarefaction analyses indicated near-saturated sampling for all samples except of S-B.1 (Additional file 1).

**Table 2:** Sample statistics including number of sequences and OTUs per sample, alpha diversity estimates and proportions of rare and abundant OTUs

	S-M.1	S-M.2	S-M.3	S-M.4	S-B.1	S-B.2	S-B.3	DS2.1	DS2.2	DS1.1	DS1.2	DS1.3
No. of filtered ciliate sequences	48,426	46,003	30,233	39,786	78,685	137,990	86,461	40,417	29,913	27,525	25,169	50,252
No. of ciliate OTUs (97%)	37	41	55	51	41	12	13	41	41	33	17	16
Effective no. of species	10.3	14.1	15.3	12.4	4.3	1.9	2.2	12.3	7.6	3.8	2.0	3.7
No. of rare OTUs	15	11	22	15	24	7	6	13	14	15	11	9
Proportion of rare OTUs [%]	41	26	40	29	60	58	46	32	33	47	69	56
No. of abundant OTUs	15	19	19	11	9	3	5	15	8	7	6	7
Proportion of abundant OTUs [%]	41	45	35	22	23	25	38	37	20	22	38	44

### Distribution of ciliate OTUs along water depth gradients and sediment layers

Observed benthic ciliate alpha diversity varied remarkably among the different sites and sediment depths. The effective number of species was on average highest at sampling site S-M and lowest at site S-B (S-M: 13 ± 1.9; DS2: 9.9 ± 2.4; DS1: 3.1 ± 0.8; S-B: 2.8 ± 1.1), whereas OTU richness was on average highest at site S-M and lowest at DS1 (S-M: 46.3 ± 7.1; DS2: 41 ± 0.0; S-B: 21.7 ± 12.9, DS1: 21.3 ± 7.5; Table 2). The number of OTUs in surface sediments along water depth was relatively stable and ranged from 33 to 41. Except of sampling site S-M, the effective number of species and the OTU richness were higher in the surface sediment samples than in the deeper layers (Table 2).

About 24% of the ciliate OTUs detected in this study were unique to one sample. No single OTU was present in all the 12 samples, whereas 20 OTUs (19%) were detected at more than six sampling sites. Abundant OTUs in the dataset accounted for 20% (DS2.2) to 45% (S-M.2), whereas rare OTUs contributed 26% (S-M.2) to 69% (DS1.2; Table 2).

**Fig. 1:**
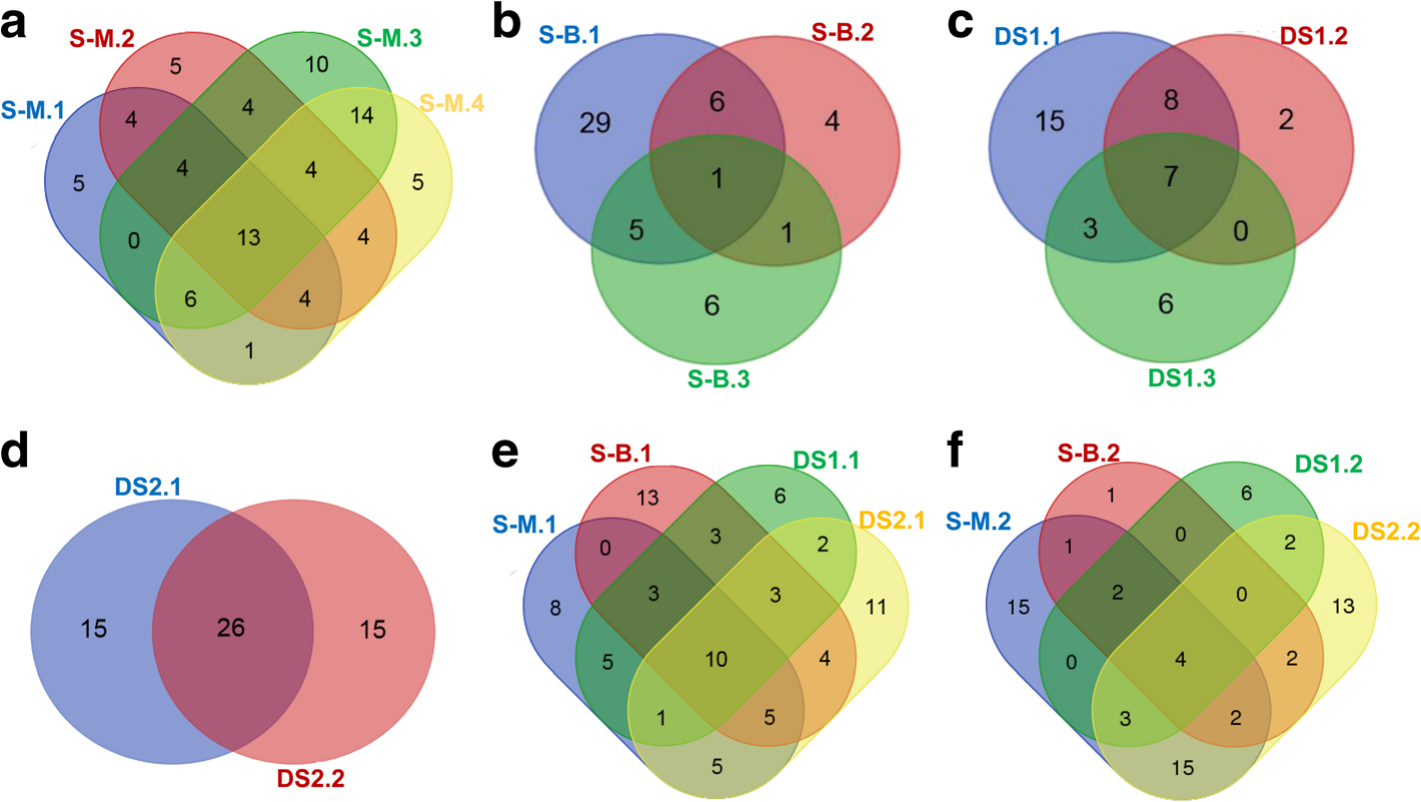
Venn diagrams displaying the number of unique and shared OTUs in different layers at sites S-M (**a**), S-B (**b**), DS1 (**c**) and DS2 (**d**), as well as in the surface layers (**e**) and in the layers of 9-10 cm depth (**f**) of different sites

At a local scale (i.e. sediment depth gradient), the major proportion of OTUs was unique to one sample and accounted for 30.1% (S-M) to 75% (S-B), whereas OTUs shared by 4 (S-M), 3 (S-B, DS1) or 2 (DS2) samples only contributed 1.9% (S-B) to 46.4% (DS2) to the ciliate OTU distribution (Fig. 1 a-d). Also on a water depth gradient, the proportion of unique OTUs was between 42.2% (Fig. 1 f) and 54.3% (Fig. 1 e) and that of shared OTUs (by 4 sites) only around 14%. 29 OTUs (78%) of the surface layer at the shallow site S-M were shared with the surface layers at sites deeper than 3800 m (Fig. 1 e).

**Fig. 2:**
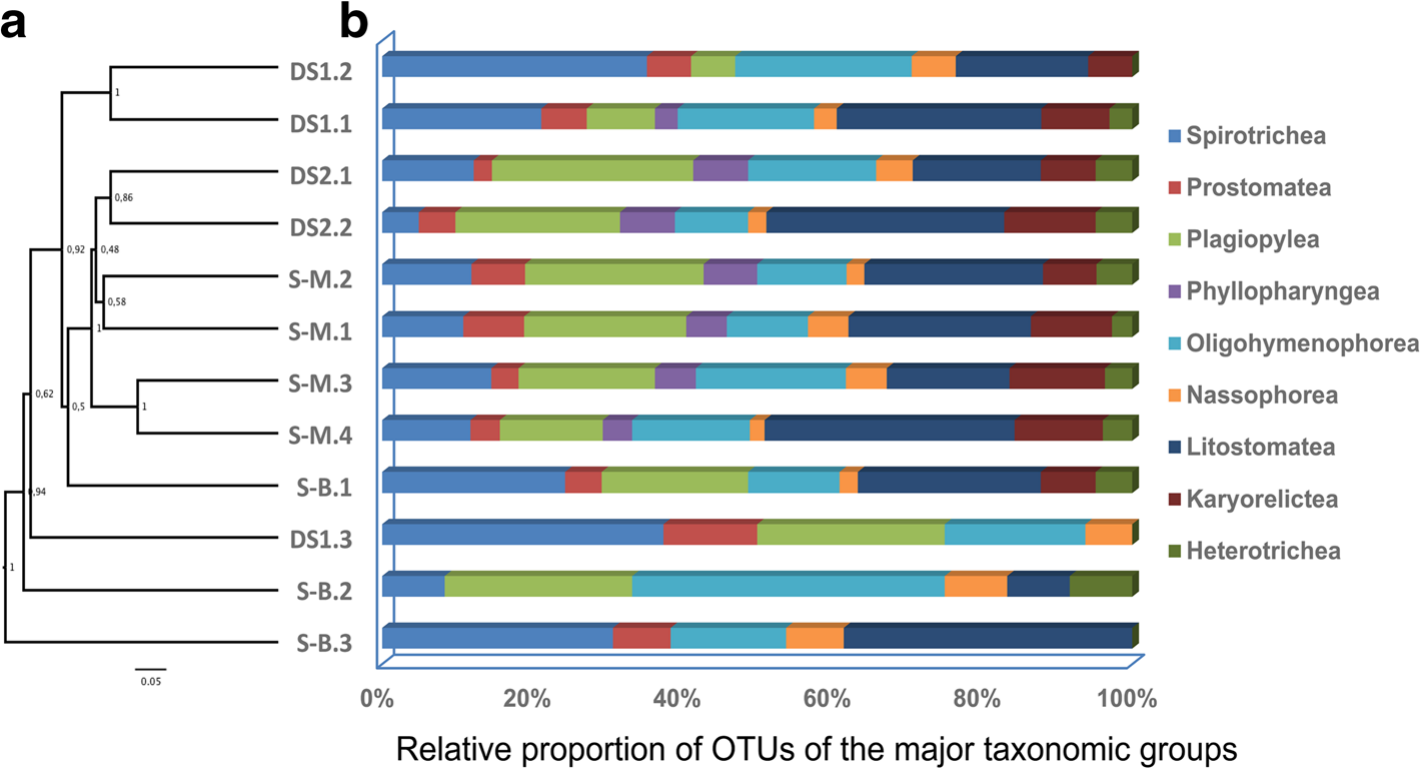
UPGMA clustering analysis based on the incidence Jaccard index (**a**), and the relative proportion of OTUs (**b**) belonging to the major taxonomic groups of ciliates detected in the 12 sediment samples

Partitioning of diversity revealed that the ciliate community structure was more similar within the upper sediment layers (0-1 and 9-10 cm) compared to the lower sediment layers (19-20 and 29-30 cm) at each site. Ciliate communities in the upper sediment layers (0-1 and 9-10 cm) of sites DS1, DS2 and S-M grouped together, respectively, with a high mean Jaccard similarity indicating a high stability of these clusters (Fig. 2 a). Ciliate communities from site S-B are distinct to the other ciliate communities and do not group into the clustering pattern (Fig. 2 a). Taking sequence abundances into account, this pattern is confirmed, although the overall grouping of the samples is slightly different (Additional file 2 a).

Ciliate diversity, i.e. OTU richness and effective number of species, and ciliate community composition in the surface sediments were not significantly correlated with any of the measured environmental parameters (Additional file 3). Additionally, changes of ciliate community composition in the deep-sea sediments were not significantly related to any combination of the environmental factors. The Mantel test showed that the pairwise community dissimilarity had no significant correlation with the geographic distance at a 350 km distance scale (*r* = −0.432, *p* = 0.83).

### Taxonomic classification of ciliate OTUs

Ciliate OTUs were assigned to 9 (out of 12) ciliate classes (Fig. 2 b; Additional file 2 b), originating from 40 families and 51 genera (Additional file 4). The classes Armophorea, Cariacotrichea and Colpoda were not detected. Most OTUs belonged to the class Litostomatea (on average 23.3% of total OTUs at each site). The Litostomatea accounted for up to 33.3% (S-M.4: 17 OTUs) of the total ciliate OTU diversity in the investigated sediment samples. However, litostomatean sequences contributed only 4.7% to the total ciliate sequence abundance (up to 15.6% in sample S-M.2; Additional file 2 b). All of these OTUs were affiliated to 8 known genera. Within the Litostomatea, sequences related to *Phialina, Loxophyllum* and *Litonotus* were most abundant (Additional file 4). No litostomatean sequences could be retrieved from sample DS1.3.

Plagiopylea were the second most diverse ciliate group, contributing on average 18.6% to the OTU diversity at each site, but 47.8% to the total sequence abundance. Plagiopylean ciliates were detected at 11 of 12 sites, with the exception of site S-B.3. The proportion of plagiopylean OTUs varied between 0 (S-B.3) and 26.8% (DS2.1: 11 OTUs) and the proportion of sequences between 0 (S-B.3) and 78% (S-B.2) (Fig. 2 b, Additional file 2 b). All OTUs/sequences were related to the anaerobic ciliate *Epalxella* (Additional file 4). The sequence similarities of these OTUs to *Epalxella* ranged between 85% and 94%.

Oligohymenophorean and spirotrichean OTUs accounted for 16% on average of all detected OTUs at each site, respectively. The proportion of OTUs affiliated to Oligohymenophorea ranged between 4.9% (S-B.3: 2 OTUs) and 20% (S-M.3: 11 OTUs) (Fig. 2 b). Oligohymenophorean sequences accounted for 35.4% of all ciliate sequences, with the lowest proportion of sequences detected in sample DS1.3 (3%) and the highest in sample S-B.3 (90%) (Additional file 2 b). All oligohymenophorean OTUs/sequences were assigned to 10 known genera and one unclassified taxon. Within this class, sequences related to *Pseudocyclidium*, *Trichodina* and *Pleuronema* were most abundant. The most cosmopolitan oligohymenophorean OTU, which could be detected in 11 of 12 samples, was closely related to *Pleuronema setigerum* with the similarity of 99% (Additional file 4).

The proportion of spirotrichean OTUs ranged between 4.9% (DS2.2) and 37.5% (S-M.3) in the different benthic ciliate communities, all of these OTUs were affiliated to 15 known genera (Additional file 4). Spirotrichean sequences contributed 8.9% to the total number of ciliate sequences, the sequence proportion accounted for up to 35.6% in sample DS1.3 (Additional file 2 b). Within this class, sequences related to the genera *Amphisiella*, *Apokeronopsis* and *Aspidisca* were most abundant. Ten of 21 OTUs, which were observed in six or more samples, belonged to Spirotrichea (Additional file 4), of which five were affiliated to the order Urostylida. The OTUs affiliated to oligotrich ciliates of *Novistrombidium orientale* and *Parallelostrombidium obesum* were detected in the down-core sediments only (Additional file 4).

Classes Karyorelictea (on average 8.8% of total number of OTUs in each sample), Prostomatea (5.3%), Phyllopharyngea (4.3%), Nassophorea (4%) and Heterotrichea (3.8%) were relatively rare (Fig. 2 b, Additional file 2 b) at all sampling sites.

### Degree of novel diversity

**Fig. 3:**
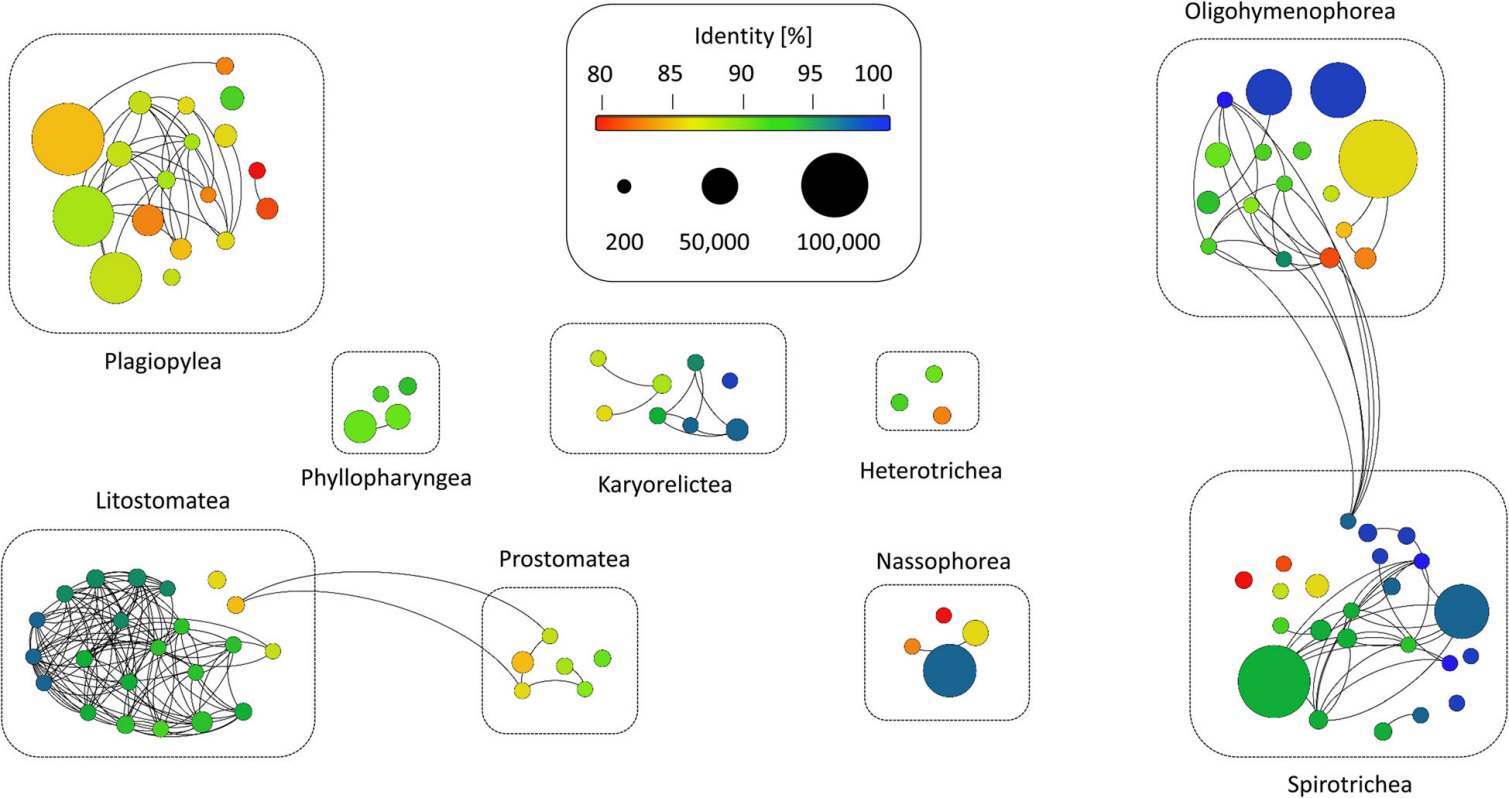
Analyses of the novel diversity within the ciliate communities in the sediments of the seamount and adjacent deep-sea plain. Each node represents one OTU, the different colors indicate the sequence similarity to a deposited reference sequence, and the size of the node indicates the number of V4 sequences. An edge weight (sequence similarity) of 90% was chosen to discriminate between the different OTUs

The observation of numerous low-identity (< 97%) OTUs in our dataset points to a high genetic novelty within the deep-sea habitats. The mean similarity of all detected ciliate OTUs to previously described sequences was only 93.25%. 73% of OTUs had a sequence similarity of less than 97% to reference sequences and 25% of the OTUs had an identity match of less than 90%. The mean similarities for each sample were relative stable and ranged between 92.86% (S-M.1) and 94.55% (DS1.1). The lowest overall mean sequence similarities, i.e. the highest degree of novel diversity occurred within the class Plagiopylea (89%). All of the 18 plagiopylean OTUs had a sequence similarity < 95%. The highest overall mean sequence similarities were observed within the classes Spirotrichea and Karyorelictea (95.7%, respectively; Fig. 3). Eleven of 23 spirotrichean OTUs and 4 of 8 karyorelictean OTUs had a sequence similarity of equal or more than 97%.

### Comparison of benthic ciliate communities from different marine habitats

OTU richness peaked in the continental shelf samples (mean OTU richness: 105.8 ± 44), followed by the intertidal samples (mean OTU richness: 93.3 ± 25) and the hydrothermal vent samples (mean OTU richness: 40 ± 21). Alpha diversity was lowest in the seamount and abyssal plain samples (mean OTU richness: 34 ± 4). Changes in alpha diversity were significant between ciliate communities from the intertidal zone (*p* = 0.01) or continental shelf (*p* = 0.01), respectively, and the communities of the seamount and abyssal plain. No significant difference was detected between the OTU richness of the seamount and abyssal plain communities and the hydrothermal vent communities.

**Fig. 4:**
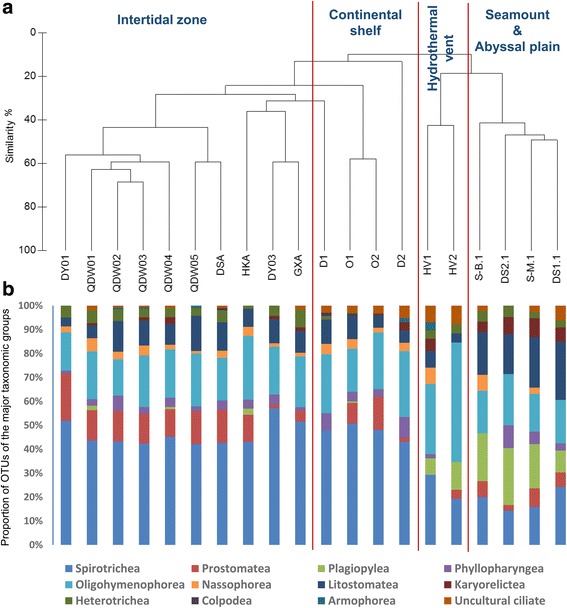
UPGMA clustering analysis based on the Sorensen-Dice values (**a**), and the relative proportion of OTUs (**b**) belonging to the major taxonomic groups of ciliates detected from the surface sediments of the intertidal zone and the continental shelf of China sea area, and the hydrothermal vent in Okinawa trough, as well as the seamount and abyssal plain in the Western Pacific Ocean

The proportion of spirotrichean OTUs was highest in the communities from the intertidal zone and continental shelf (Fig. 4). Almost no Plagiopylea ciliate was detected in the coastal samples, whereas Plagiopylea was one of the most diverse ciliate group in the seamount, abyssal plain, and hydrothermal vent samples (Fig. 4). The proportion of oligohymenophorean OTUs was the highest in the communities of the hydrothermal vent samples. UPGMA clustering based on the Sorensen-Dice similarity coefficient between the ciliate communities revealed two large clusters, separating intertidal and offshore communities from the deep-sea seamount, abyssal plain and hydrothermal vent communities (Fig 4). ANOSIM analyses confirmed this observation showing a significant disparity among different habitat types (*r* = 0.92; *p* = 0.001).

## Discussion

### Ciliate diversity and their distribution along the water depth gradient

Our study revealed the diversity of ciliates along a wide water depth gradient (813-4566 m) in the sediments from a seamount and an adjacent abyssal plain in the Western Pacific Ocean. Although sample saturation was reached for all but one sample, we found a notably lower number of distinct ciliate OTUs (33-41 OTUs at each site) in the surface sediment samples analyzed in this study compared to a previous molecular diversity survey of protists in deep-sea sediments (68-172 ciliate OTUs at each site) [7]. This could be partially explained by methodological differences, such as the use of another barcode marker (V9 vs. V4 region of SSU rDNA) and/or the deletion of singletons (OTUs containing only one sequence) in our study, which aimed to improve specificity at the cost of a possible loss in sensitivity. Instead, our results corroborate well with a microscopy study [14], suggesting a lower diversity of ciliates in deep-sea sediments compared to that in sediments from an intertidal zone and a continental shelf (e.g. 58-115 OTUs in the Yellow Sea and the East China Sea [21, 22]; 77-365 OTUs in the European coastal regions [23]). However, if water depth is deep enough, e.g. 813 m or more than 3800 m, it seems that water depth has little to no effect on the benthic ciliate OTU richness. This is different from the conclusion of the investigation in coastal sediments, where water depth overrides both geographic distance and environmental heterogeneity in determining the community composition of protists [16]. Likewise, ciliate biomass and the number of species showed a significantly negative correlation with water depth in the offshore sediments of the Yellow Sea [24]. However, little variation was observed in microeukaryotic communities from abyssal depths ranging between 5033 m and 5655 m [25]. Likewise, using the data provided by the study of Pawlowski et al. for water depths ranging between 2292 and 6326 m, we could not detect a significant correlation between the benthic ciliate OTU richness and water depth [7]. This indicates that water depth is likely more important on influencing the distribution of microbial eukaryotes in coastal sediments than in deep-sea sediments.

In our study, other factors than water depth might be more relevant for the structuring of ciliates in deep-sea sediments, e.g. oxygen saturation, which is a decisive variable for shaping protist community in shallow water sediments [17–20]. Unfortunately, this parameter could not be measured in our study.

Sediment patchiness at the deep-sea bottom is extremely high [26], possibly explaining the high dissimilarity of ciliate communities between all samples and the low number of shared OTUs between samples. Since rarefaction analyses confirm near-saturated sampling profiles for all but one sample, undersampling is no reason for the observed differences in ciliate communities.

Previous studies mainly focused on high taxonomic levels, such as the phylum level, when investigating the proportion of ciliates in a protist community (e.g. [7]). However, there is no detailed information on the assemblage of benthic ciliates in deep sea environments, except of the deep-sea hydrothermal vents [27] and cold seeps [28]. In our study, we, for the first time, revealed that the pattern in the sediments of a seamount and the adjacent abyssal plain is quite different from that observed in intertidal zones and offshore areas, where spirotrichs are predominant in terms of relative abundance and species richness [20–22, 29]. By contrast, in the sediments of the seamount and the abyssal plain, Plagiopylea ciliates were obviously abundant and diverse*.* The high sequence contribution implies that they might have contributed a high biomass in the ciliate community in these habitats [30]. Plagiopylea ciliates have generally been found in high sulfide, anoxic sediments [31]. Previous studies indicate that these ciliates are abundant in sediments from the hydrothermal vents [22, 27]. Our study, therefore, extends the knowledge on the distribution of Plagiopylea ciliates, and reveals their potential importance in the benthic ciliate communities of seamounts and abyssal plains. However, some ciliate genera, like *Pleuronema,* which contains more than 20 morphotypes reported from various geographical locations of intertidal and coastal zones [32], were found to be widely distributed along the pronounced range of water depths, revealing their cosmopolitan character.

Regarding the proportion of rare OTUs in the sediments of a seamount and adjacent abyssal plain, there was no obvious shift along the water depth gradient. The proportion of rare OTUs detected in our study was obviously lower than what is known from intertidal and coastal sediments [21]. Rare taxa can be considered as a seed bank of genetic resources, and are hypothesized to include ecologically redundant taxa that could increase in abundance following environmental perturbation to maintain continuous ecosystem functioning [33, 34]. Compared to coastal zones, deep-sea benthic habitats are relatively stable environments, explaining the observed lower proportion of rare taxa.

### Vertical distribution of ciliates in the deep-sea sediments

In intertidal sediments, previous studies of benthic protists have shown that the abundance and species richness of protozoa were highest at the top 1 to 2 cm [17, 18]. Likewise, ciliate abundance and biomass in the 0-2 cm layers of coastal sediments accounted for 77% and 81%, respectively [24]. Several species, however, were also found to live below 5 cm, even in 8 cm sediment depth [17, 18]. Previous studies showed that the respective proportions of ciliate abundance were about 23% in the 3–8-cm layers [24]. Our study detected a high diversity of ciliate sequences in 20 cm sediment depth. When working with DNA, this is certainly no proof for the presence of living and active ciliate species, as DNA can be preserved in marine sediments over time. However, ciliates are very well adapted to a variety of different environment types, being able to not only tolerate anoxia, high salt or hydrogen sulfide concentrations or high pressure but also combinations of those [22, 27, 28]. It, therefore, would not be surprising if ciliates evolved to live in this water and sediment depths, especially in the presence of large bacterial populations in these deep sediment spheres [35, 36], which could serve as food source for heterotrophic ciliates.

The observation of OTUs affiliated to oligotrich ciliates in the deep layer of the deep-sea sediments confirmed a fraction of ciliate DNA sequences which likely do not belong to living organisms, but represent either extracellular DNA or cysts of planktonic species. The deep-sea floor, therefore, appears as a global DNA repository, which preserves molecular information of organisms living in the sediments, as well as in the overlying water column [37]. In contrast, OTUs, which are found in RNA surveys, were more likely active species [38, 39]. Thus, RNA surveys should provide a better representation of in situ protist biomass and diversity. Clearly, in the future, the analyses of deep-sea RNA will be helpful to identify metabolically active organisms.

### High novelty of ciliate genetic diversity

A high degree of genetic novelty was uncovered within the ciliates from the sediments of a seamount and the adjacent abyssal plain, although ciliates are considered as the best-known group within the protists. The application of high throughput DNA sequencing contributed substantially to the detection of a broader protist diversity [7, 40]. The extremely high molecular diversity largely exceeded the one detected by morphological methods. Thus, there might be some artifacts resulting from limitations of the techniques. Previous studies have indicated that protists are well known for having extremely high rDNA copy numbers [41, 42], which might result in a high polymorphism of SSU rDNA in a single cell and lead to the overestimation of molecular diversity in environmental samples. Gong et al. [41] showed that the minimal similarity between two copies in a single ciliate cell was 99.1%. The commonly used cutoff of 97% for creating OTUs based on the ciliate V4 fragment of their 18S rDNA is very well analyzed and established and excludes the effect of intragenomic variations [23, 43–46].

Moreover, there is still an obvious gap between the obtained sequences and the reference databases, which are used for taxonomic assignment of the sequences. The ciliate class Plagiopylea is such an example. Only one species, i.e. *Epalxella antiquorum*, within the order Odontostomatida and a total of eight plagiopylean species had their 18S rDNA sequences deposited in the NCBI database (searched on July 4th, 2017). The lack of reference sequences in the database resulted in all plagiopylean OTUs having a sequence similarity of less than 95% to the closest reference sequence. Additionally, even the most abundant plagiopylean OTU had only a sequence similarity of less than 90% to the reference sequences. More efforts in the isolation, cultivation and description of protists are necessary to link the environmental sequences to the real protist inventory [23, 47]. The design of novel species-specific primers and probes based on the retrieved sequences will also help to identify the target species by molecular techniques [48, 49]. In addition, different ciliate species were not equally distributed along a vertical sediment gradient and a large proportion of unknown ciliate diversity was found in deeper sediment layers. In the future, a more adequate sampling strategy is needed, which could maximize the number of different deep-sea habitats, and thus, detect more new ciliate species.

## Conclusions

Our data point to obvious variations in ciliate communities along a sediment depth gradient in a seamount and its adjacent abyssal plain in the tropical Western Pacific Ocean and reflect the heterogeneity and diversity of benthic habitats. Water depth occurred as a less important factor impacting ciliate distribution in deep-sea sediments unlike observed for benthic ciliates in shallow seafloor habitats. Furthermore, our results indicate that ciliate diversity is not as well known as previously assumed and further efforts have to be made towards the identification of ciliate species. In this respect, deep-sea sediments appear as a hotspot for capturing and investigating novel ciliate species.

## Methods

### Study sites and sampling

**Fig. 5:**
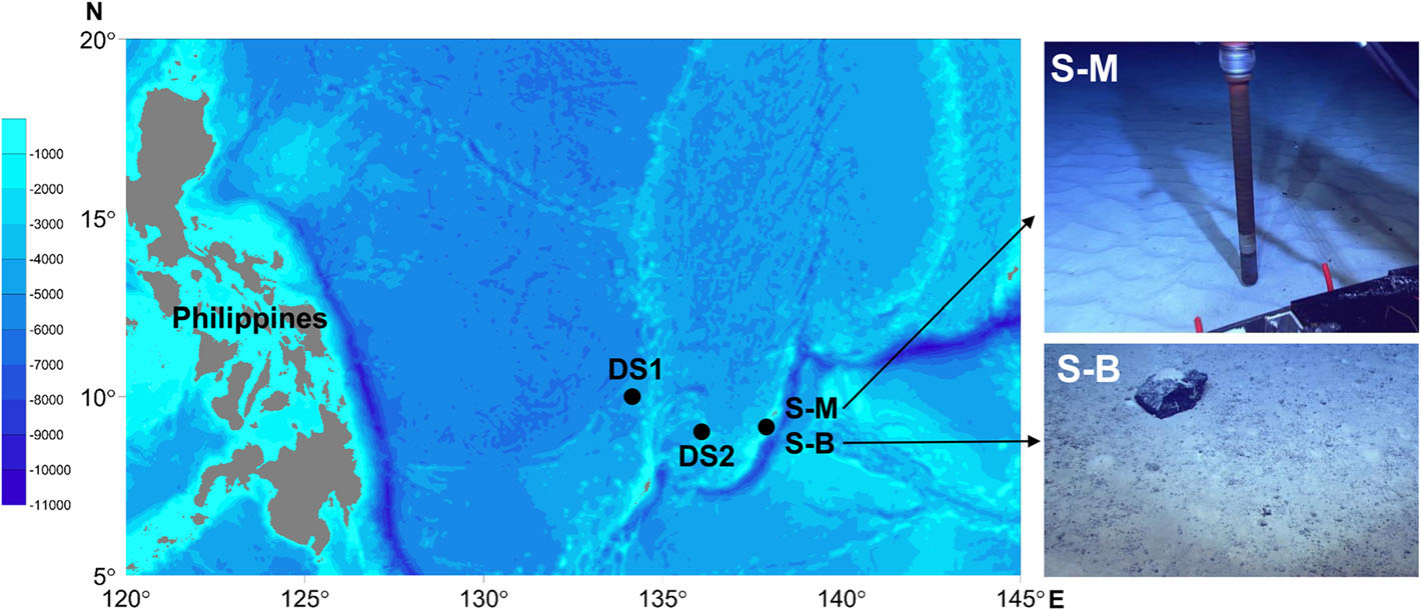
Location of the four sampling sites in the tropical Western Pacific Ocean. Site S-M is located in the middle of a seamount adjacent to the Yap Trench at a water depth of 813 m, and site S-B at the foot of the seamount at a water depth of 3812 m. Sampling sites DS1 and DS2 are both located in the adjacent deep-sea plain with depths of 4042 m and 4566 m, respectively. Images on the right illustrate the different sediment types observed at the seamount

Sediment samples were collected from four sites in the tropical Western Pacific Ocean (Fig. 5). Among these, site S-M is located at the middle of a seamount adjacent to the Yap Trench at a water depth of 813 m, and site S-B at the foot of the seamount at a water depth of 3812 m (Table 1). Sediment samples of S-M and S-B were collected using a push-corer operated by the remotely operated vehicle (ROV) “Discovery” onboard the *R/V KEXUE* in December 2014. Sampling sites DS1 and DS2 are located in the adjacent abyssal plain with water depths of 4042 m and 4566 m, respectively (Fig. 5). Sediment samples of DS1 and DS2 were taken using a 0.25-m^2^ modified Gray-O’Hara box corer in December 2014. Sites S-M and S-B are about 20 km distant from each other, and about 200 km apart from site DS2 and about 350 km apart from site DS1. Site DS1 is about 170 km distant from site DS2.

Four layers of the sediment cores (0-1 cm, 9-10 cm, 19-20 cm and 29-30 cm; labelled as 1, 2, 3 and 4, respectively) were sampled at site S-M, three layers (0-1 cm, 9-10 cm and 19-20 cm; labelled as 1, 2 and 3, respectively) at sites S-B and DS1, and two layers (0-1 cm, 9-10 cm; labelled as 1 and 2, respectively) at site DS2.

For each site and each layer, three replicate samples, each containing about 15 g sediments, were taken. Samples were stored at −80 °C for DNA extraction, at −20 °C for examination of total organic carbon (TOC), and at 4 °C for grain size measurements. TOC was determined in a Vario TOC cube (Elementar, Germany). Grain size analyses were performed using a Laser Diffraction Particle Size Analyzer (Cilas 940 L).

### DNA extraction, PCR amplification and high-throughput sequencing

Environmental DNA was extracted from 0.3 g sediment of each replicate sample (a total of 3 DNA samples for each sediment sample) using the Power Soil DNA isolation kit (MoBio Laboratories, USA) according to the manufacturer’s protocol. Ciliate sequences were amplified by a nested PCR approach [50], using ciliate-specific 18S rRNA gene primers in the first reaction. The second PCR reaction employed a primer set specific for the hypervariable V4 region, which was included in the first PCR product. To minimize PCR errors, we used the PrimeSTAR GXL DNA High Fidelity Polymerase (TAKARA BIO INC., Japan). Three products from each sample were pooled.

Sequencing libraries were constructed using the NEB Next® Ultra™ DNA Library Prep Kit for Illumina (NEB, USA). Quality of the libraries was assessed with an Agilent Bioanalyzer 2100 system. Finally, libraries were sequenced on an Illumina MiSeq platform, generating 300-bp paired-end reads. The ciliate sequence reads have been deposited at the National Center for Biotechnology Information (NCBI) Sequence Read Archive under the accession number SRP101585.

### Sequence data processing

Paired-end reads were merged and then filtered. Quality filtering of the raw sequences was performed according to the QIIME quality control process [51]. Afterwards, a set of unique sequences was identified and the number of occurrences for each sequence was recorded by UPARSE software, followed by discarding all singleton sequences [52]. A sequence similarity of 97% was used to delineate ciliate OTUs [43–46] by the UPARSE-OTU algorithm. Representative sequences from each OTU were extracted and subjected to the basic local alignment search tool (BLAST) analyses against the Silva database (v. 123) as implemented in the QIIME pipeline (v. 1.9.0) [51].

In each sample, OTUs were defined as rare when their number was equal to or less than 0.1% of all the sequences in the sample, and abundant, when their number was equal to or more than 1% of all the sequences in the sample [34, 53].

### Statistical analysis

Statistical analyses were conducted in R using the vegan and fossil packages, unless stated otherwise. Rarefaction analyses were conducted in order to investigate the degree of sample saturation. Alpha diversity for each sample was calculated by the exponential of the Shannon index H′ (exp(H′), effective number of species) [54]. After conversion, the effective numbers of species lets us avoid the serious misinterpretations spawned by the nonlinearity of the Shannon index H′ [54]. Additionally, the ciliate OTU richness was determined as the number of OTUs in each sample. Spearman’s coefficient was used to relate ciliate alpha diversity and the environmental parameters, including water depth, TOC, the median grain size and the proportions of sand, silt and clay.

Partitioning of diversity (beta diversity) was investigated by an unweighted pair-group method with arithmetic means (UPGMA) cluster analysis based on the incidence Jaccard index. Stability in cluster analysis was evaluated by bootstrap resampling (100 times) using the clusterboot function in the fpc package of R [55]. Here, the Jaccard similarities of the original clusters to the most similar clusters in the resampled data were computed. The mean over these similarities was then used as an index of the stability of a cluster. Generally, “highly stable” clusters should yield average Jaccard similarities of 0.85 and above. Clusters with a Jaccard similarity value smaller or equal to 0.5 were considered as a “dissolved cluster” [56]. Prior to alpha and beta diversity analyses, the OTU table was randomly subsampled to the lowest number of sequences present in a sample (*n* = 25,169).

RALATE function in PRIMER v6 (Plymouth Marine Laboratory, UK) was applied to calculate Spearman’s correlation coefficients between the ciliate community composition and the environmental parameters. The BIOENV function in PRIMER v6 was used to calculate the correlation between the ciliate community and the environmental similarity matrix to determine the variables that best explain variation in the ciliate community.

The significance of the relationship between community Bray-Curtis dissimilarity and geographical distance (distance in kilometers between pairs of sites) within the detected distance-decay extent was assessed by a Mantel test for each data set. The Mantel tests were performed using the vegan package in R [57].

### Identification of novel diversity

Identification of novel diversity followed the description of Filker et al. 2015 [47]. Briefly, representative sequences of all detected OTUs were aligned with Seaview v.4.6.1 using “clusto” [58]. Based on these alignments, pairwise similarities were calculated (custom script) and used for network construction in R (“igraph” package) [59]. The resulting network was visualized and modified with GEPHI v.0.9.1. according to the OTU taxonomic affiliation and the Blast Hit values [60]. In the network, two nodes were connected by an edge if they shared a sequence similarity of at least 90%.

### Comparison of benthic ciliate communities from different marine habitats

We collected a set of publicly available data that were related to high throughput sequencing of the ciliate V4 fragment of 18S rDNA. These sequences were obtained from the surface sediments of the intertidal zone (SPR068269, [21]), the continental shelf of the Yellow Sea and East China Sea, and the hydrothermal vents in Okinawa Trough (SPR064020, [22]). Sequence data were processed as the protocol mentioned above. The significance of differences in the OTU richness among different habitats were estimated with Tukey’s HSD test. To investigate the partitioning of ciliate diversity, the abundance-based OTU table was transformed into a presence/absence table prior to calculating Sorensen-Dice indices. Sorensen-Dice values were then used for UPGMA cluster analyses. The ANOSIM function in PRIMER v6 was used to test the differences among different habitats. ANOSIM provides an R-statistic to evaluate the dissimilarity of groups, thus, groups are dissimilar if R-statistic is close to 1. Prior to alpha and beta diversity analyses, the OTU table was randomly subsampled to the lowest number of sequences present in a sample (*n* = 22,000).

## Additional files

Additional file 1: Rarefaction curves for all samples under study. (TIFF 3182 kb)

Additional file 2: UPGMA clustering analysis based on the Bray-Curtis similarity coefficient (a) and the relative proportion of sequences (b) related to the major taxonomic groups of ciliates detected in the 12 sediment samples. (TIFF 713 kb)

Additional file 3: Spearman’s rank correlation coefficients between ciliate alpha diversity in the surface layer sediments and the environmental parameters. *P*-value <0.05 are considered as significant. (DOCX 14 kb)

Additional file 4: OTU-table displaying, for each sample, the number of sequences per OTU, the BLAST similarity of the representative sequence of each OTU to the reference sequence and the taxonomic assignment of the representative sequence. (XLSX 18 kb)

## Abbreviations

BLAST: Basic local alignment search tool
MGS: Median grain size
OTU: Operational taxonomic unit
ROV: Remotely operated vehicle
TOC: Total organic carbon
UPGMA: Unweighted pair-group method with arithmetic means
