# Supplementary figures and images for: Ciliate diversity and distribution patterns in the sediments of a seamount and adjacent abyssal plains in the tropical Western Pacific Ocean
Source: BMC Microbiol. 2017 Sep 12;17:192. doi: 10.1186/s12866-017-1103-6 (PMC5596958; doi:10.1186/s12866-017-1103-6)

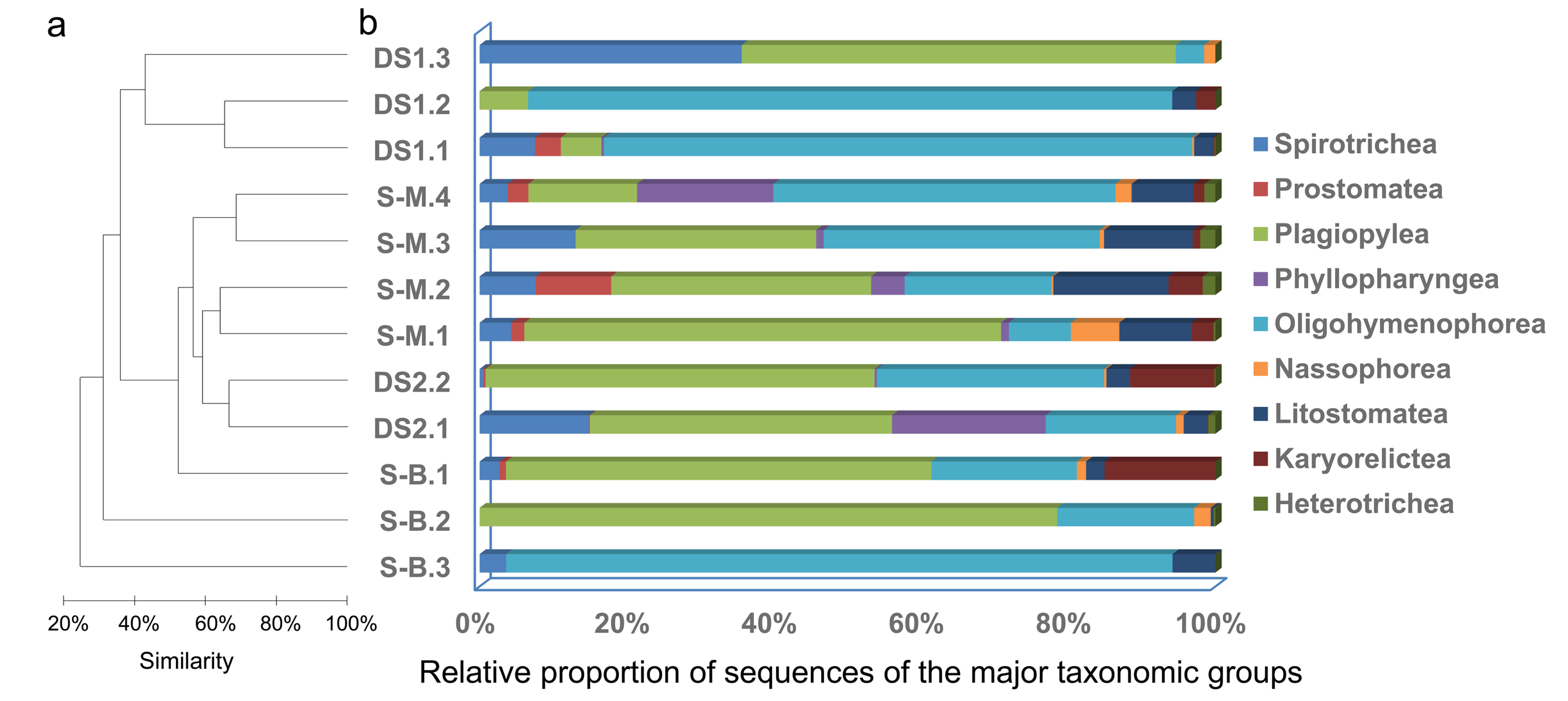

Supplement: Supplementary file 2 — UPGMA clustering analysis based on the Bray-Curtis similarity coefficient (a) and the relative proportion of sequences (b) related to the major taxonomic groups of ciliates detected in the 12 sediment samples. (TIFF 713 kb) [file 12866_2017_1103_MOESM2_ESM.tif]
