# Supplementary material for: Ciliate diversity and distribution patterns in the sediments of a seamount and adjacent abyssal plains in the tropical Western Pacific Ocean
Source: BMC Microbiol. 2017 Sep 12;17:192. doi: 10.1186/s12866-017-1103-6 (PMC5596958; doi:10.1186/s12866-017-1103-6)
Supplement: Supplementary file 3 — Spearman’s rank correlation coefficients between ciliate alpha diversity in the surface layer sediments and the environmental parameters. P-value <0.05 are considered as significant. (DOCX 14 kb) [file 12866_2017_1103_MOESM3_ESM.docx]

**Additional file 3** Spearman's rank correlation coefficients between ciliate alpha diversity in the surface layer sediments and the environmental parameters. P-value < 0.05 are considered as significant.

|  | OTU Richness | | Effective species number | | ciliate community composition | |
| --- | --- | --- | --- | --- | --- | --- |
|  | r | p | r | p | r | p |
| Water Depth | 0.63 | 0.37 | 0.20 | 0.92 | -0.43 | 0.92 |
| Total organic Carbon | 0.32 | 0.68 | 0.40 | 0.75 | -0.14 | 0.71 |
| Median grain Size | -0.95 | 0.05 | -0.4 | 0.75 | -0.83 | 0.91 |
| Sand | -0.63 | 0.37 | -0.2 | 0.92 | -0.83 | 0.93 |
| Silt | 0.63 | 0.37 | 0.20 | 0.92 | -0.83 | 0.92 |
| Clay | 0.63 | 0.37 | 0.20 | 0.92 | -0.82 | 0.91 |
